# Supplementary material for: SMCHD1 and LRIF1 converge at the FSHD-associated D4Z4 repeat and LRIF1 promoter yet display different modes of action
Source: Commun Biol. 2023 Jun 28;6:677. doi: 10.1038/s42003-023-05053-0 (PMC10307901; doi:10.1038/s42003-023-05053-0)
Supplement: Supplementary file 2 — Supplemental Information [file 42003_2023_5053_MOESM2_ESM.pdf]

Supplementary Information

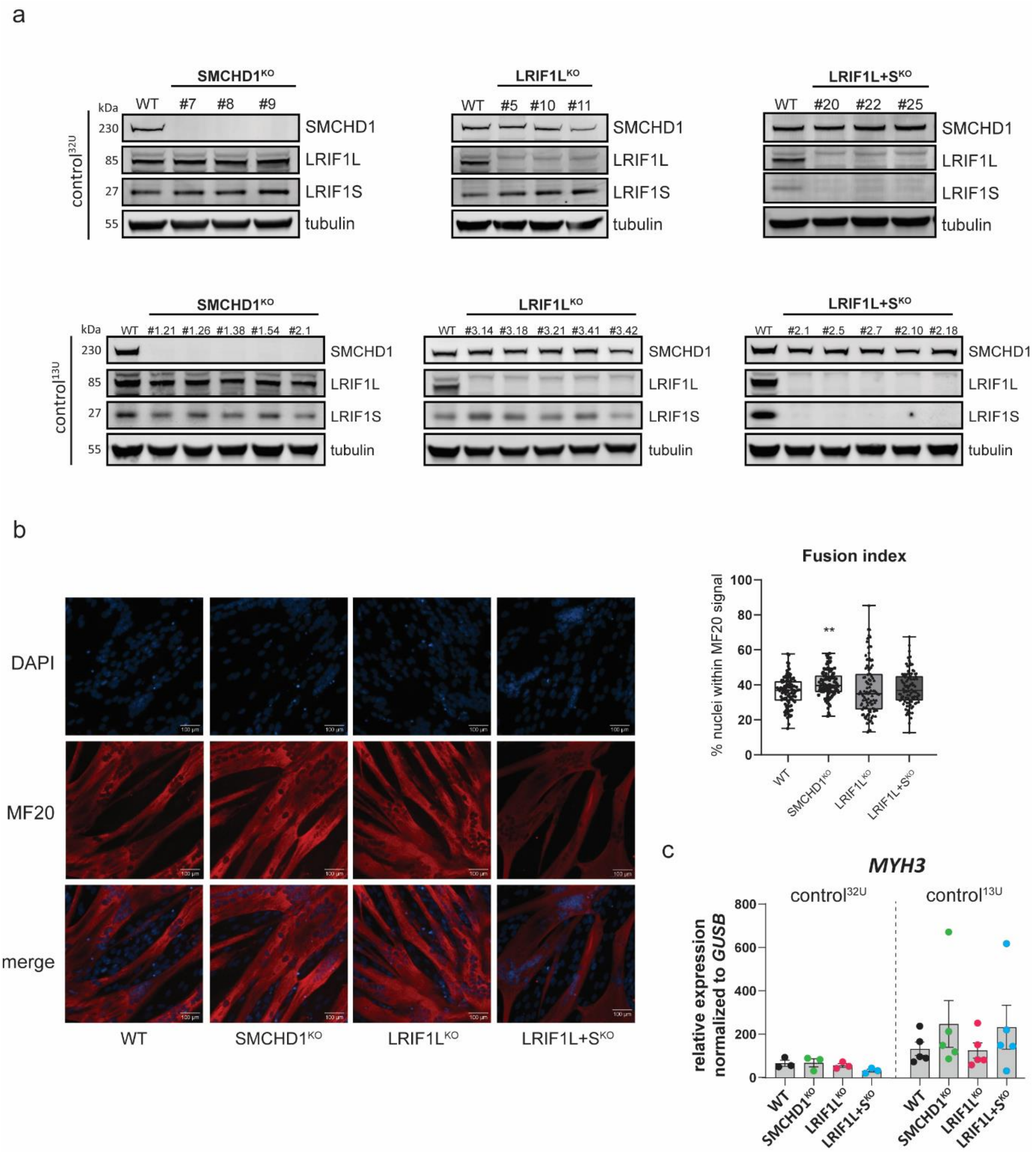

Supplementary Figure 1. Characterization of knockout clones. A) Western blot confirmation of successful SMCHD1 and LRIF1 knockouts in control^{32U} and control^{13U} myogenic cell lines. Tubulin was used as a loading control. **B)** MYH1E staining (in red) of one WT and one clone of each knockout condition of the control^{32U} cell line. Nuclei are counterstained with DAPI (in blue). Merged images show overlay of DAPI and MYH1E staining. Scale bar is 100 µm. Fusion index (=number of nuclei inside myotubes as a percentage of the total number of nuclei) was calculated for each clone of control^{32U} line that is depicted in A). Box represents 25th to 75th percentile and line represents a median value of all fusion indexes calculated from 100 images per clone, totalling on average to 10,000 nuclei positions analysed per clone. Statistical significance between WT and KO groups was calculated by one-way ANOVA with Dunnett's post hoc test (**p<0.01). **C)** RT-qPCR a myogenic marker *MYH3* in differentiated WT and knockout clones derived from respective control cell lines. Bars represent mean ± SEM. Each dot represents one clone.

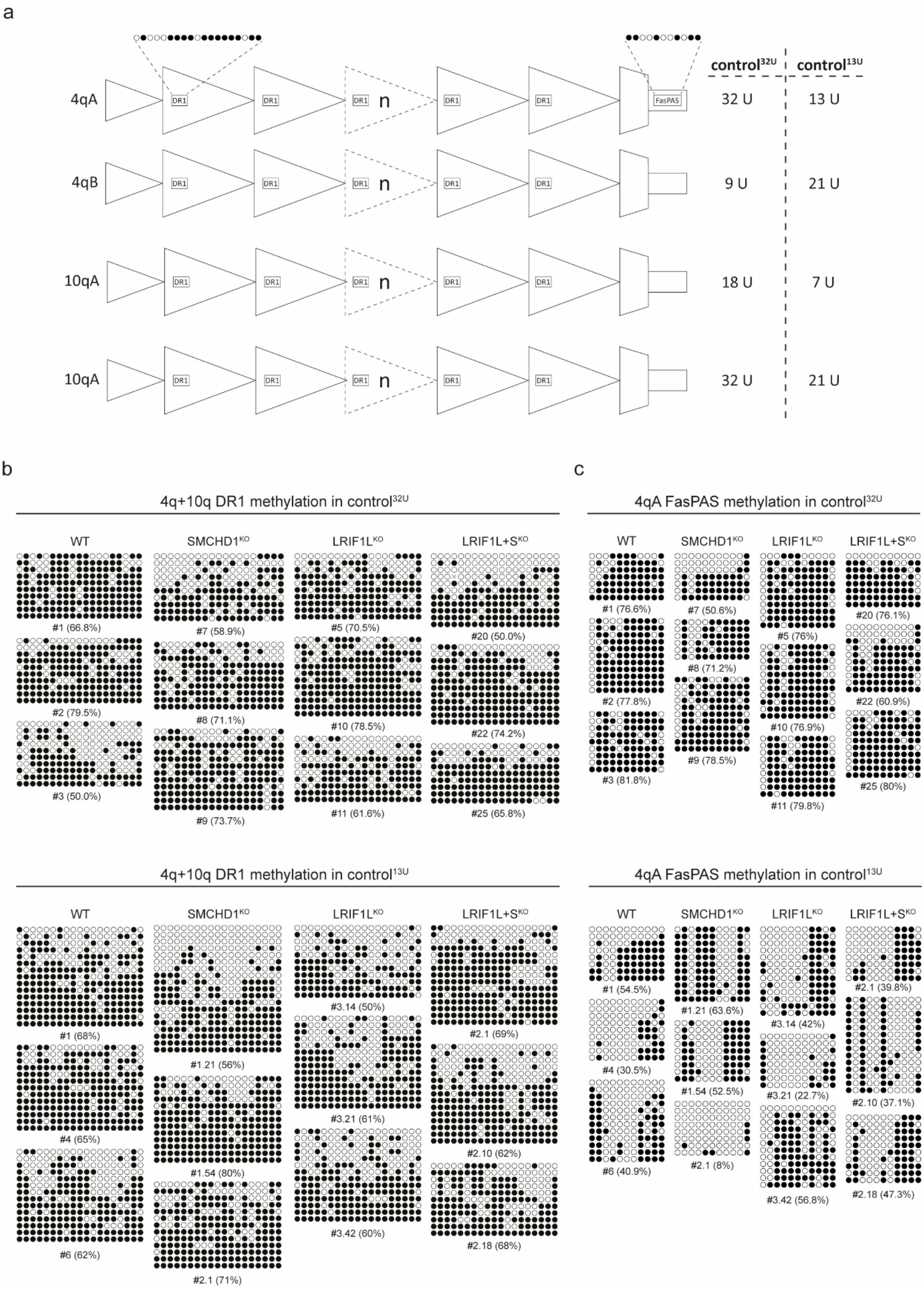

Supplementary Figure 2. Methylation of individual CpGs in D4Z4 DR1 and FasPAS regions is unchanged in different immortalized myoblast KO clones related to Figure 2A and 2B. A) Schematic representation of 4q and 10q D4Z4 allele genotypes in control^{32U} or control^{13U} cell lines together with

amplified DR1 and FasPAS regions for monitoring DNA methylation. Note that DR1 region is a multicopy locus present on both 4q and 10q D4Z4 alleles whereas FasPAS region is a single copy locus only present downstream of 4qA specific D4Z4 repeat (i.e. *DUX4* expressing allele). **B)** Lollipop representation of DR1 site methylation in different clones derived from control^{32U} or control^{13U} cell line (three independent clones per genotype). Full circles represent methylated CpGs, and open circles represent unmethylated CpGs. The mean methylation of each clone is calculated in the open brackets below each lollipop plot and plotted in Figure 2A. **C)** Lollipop representation of FasPAS site methylation in different clones derived from control^{32U} or control^{13U} cell line (three independent clones per genotype). Full circles represent methylated CpGs, and open circles represent unmethylated CpGs. The mean methylation of each clone is calculated in the open brackets below each lollipop plot and plotted in Figure 2B.

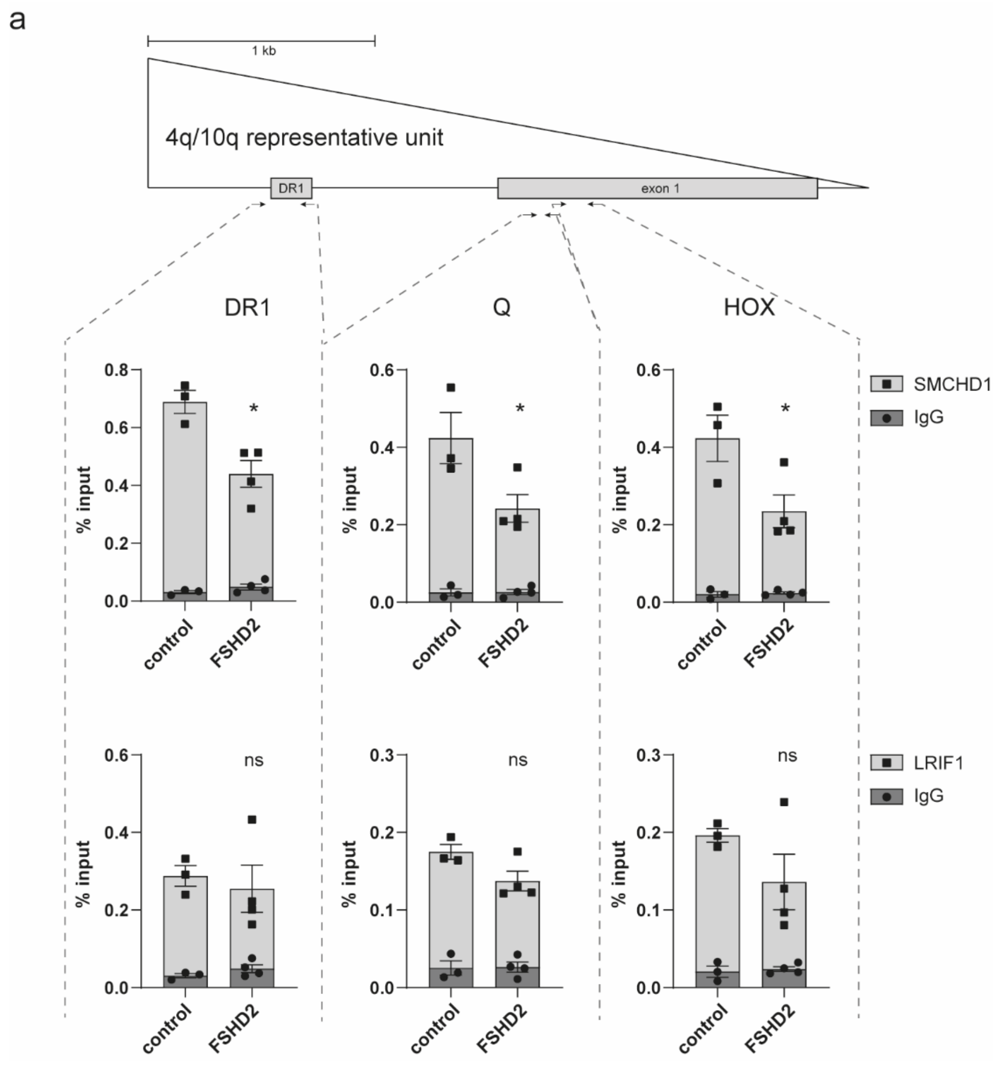

Supplementary Figure 3. SMCHD1 and LRIF1 enrichment at D4Z4 in primary control and FSHD2 myoblasts. A) SMCHD1 and LRIF1 ChIP-qPCR in different control (n = 3, lines: 1926, 2333, 2417) and FSHD2 (n = 4, lines: 2338, 2440, 2413, 2337) primary myoblasts. Schematic of one D4Z4 unit and the position of three regions within D4Z4 examined by ChIP-qPCR is indicated. Bars and whiskers represent mean ± SEM. Isotype-specific IgG was used for background control. Statistical significance between control and FSHD2 group was calculated by Student's t-test (*p<0.05).

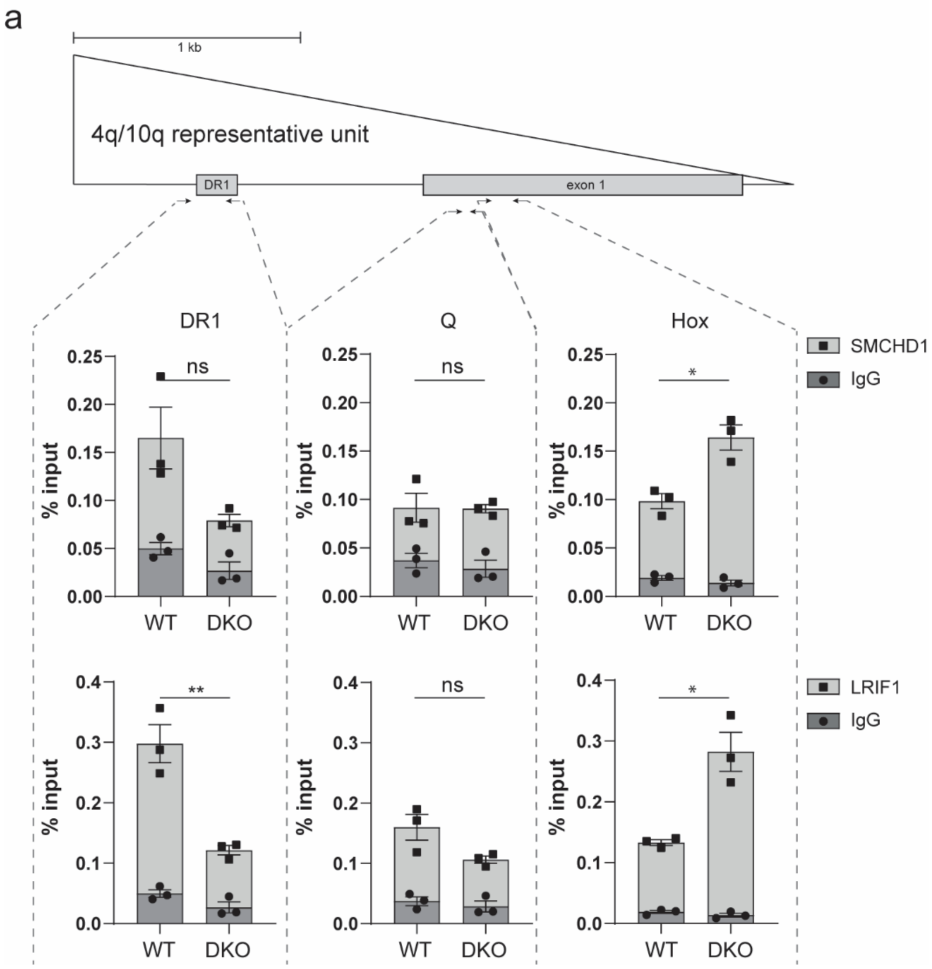

Supplementary Figure 4. SMCHD1 and LRIF1 binding to D4Z4 is reduced in HCT116 DKO cells. A) SMCHD1 and LRIF1 ChIP-qPCR in HCT116 WT and DKO cells. Schematic of one D4Z4 unit with the position of the three regions within D4Z4 examined by ChIP-qPCR are indicated (DR1, Q, HOX). Bars and whiskers represent mean ± SEM of three experiments. Isotype specific IgG was used for background control. Statistical significance was calculated with an unpaired t-test (**p<0.01,*p<0.05, ns – not significant).

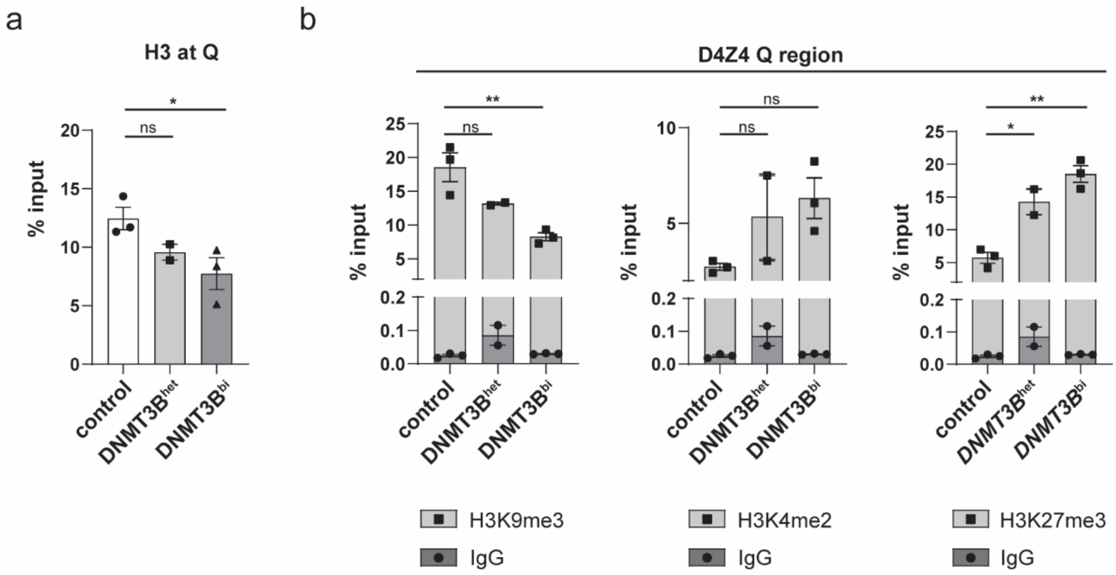

Supplementary Figure 5. Histone mark profiles in primary fibroblasts carrying heterozygous (*DNMT3B*^{het}) or biallelic *DNMT3B* mutation (*DNMT3B*^{bi}) resembles those reported in FSHD2 cases due to *SMCHD1* or *LRIF1* mutations. A) H3 ChIP-qPCR of the D4Z4 Q region in primary control fibroblasts (n = 3, lines: 2374, 2417, 2397) or fibroblasts carrying either heterozygous *DNMT3B* mutation (n = 2, lines: v294, b974) or biallelic *DNMT3B* mutations (n = 3, lines: GM08714, Rf614, Rf699.3). Bars and whiskers represent mean ± SEM. Isotype specific IgG was used for background control. **B)** ChIP-qPCR of selected histone marks at the D4Z4 Q region in the same primary fibroblast sets as in A). Bars and whiskers represent mean ± SEM. Isotype specific IgG was used for background control. Statistical significance was calculated by one-way ANOVA with Dunnett's post hoc test (**p<0.01, *p<0.05, ns – not significant).

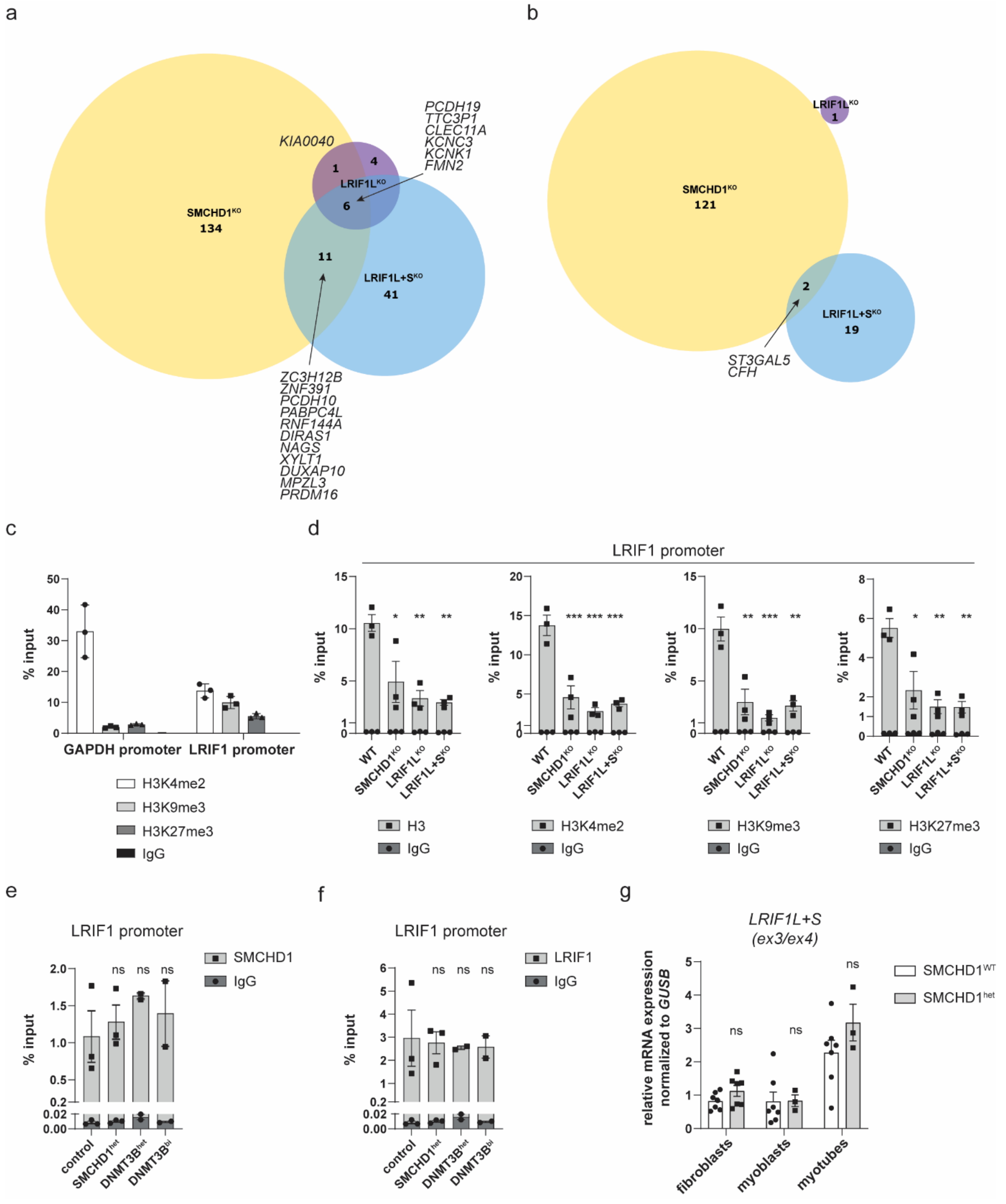

Supplementary Figure 6. ***LRIF1*** **expression is sensitive to somatic LRIF1 and SMCHD1 gene dosage perturbations. A)** Venn diagram of differentially upregulated genes overlapping between the knockout conditions. **B)** Venn diagram of differentially downregulated genes overlapping between the knockout conditions. **C)** Selected histone marks ChIP-qPCRs of *GAPDH* and *LRIF1* promoter in WT control^{32U} clones. Bars and whiskers represent mean ± SEM (ns = 3). **D)** H3 and selected H3-coupled marks ChIP-qPCR of the LRIF1 promoter in WT and different control^{32U} knockout conditions. Bars and whiskers represent mean ± SEM (ns = 3). Isotype specific IgG was used for background control. Statistical significance was calculated by one-way ANOVA with Dunnett's post hoc test (***<0.001,

p<0.01, *p<0.05). **E) SMCHD1 ChIP-qPCR of the LRIF1 promoter in primary control fibroblasts (n = 3, lines: 2524, 2397, 2333) and fibroblasts carrying either a heterozygous *SMCHD1* mutation (n = 3, lines: 2440, 2337, 2332), a heterozygous *DNMT3B* mutation (n = 2, lines: v294, b974) or biallelic *DNMT3B* mutations (n = 2, lines: Rf699.3, Rf286.3). Bars and whiskers represent mean ± SEM. Isotype specific IgG was used for background control. Statistical significance was calculated by one-way ANOVA with Dunnett's post hoc test (ns – not significant). **I)** LRIF1 ChIP-qPCR of the *LRIF1* promoter in the same samples as in H)**.** Statistical significance between WT and mutant groups was calculated by one-way ANOVA with Dunnett's post hoc test (ns – not significant). **J)** RT-qPCR of *LRIF1* in different control and SMCHD1 haploinsufficient primary cell lines (fibroblasts, myoblasts or differentiated myotubes). Bars and whiskers represent mean ± SEM. Each dot represents cell line derived from a unique individual. Statistical significance between control and $SMCHD1^{het}$ group was calculated with an unpaired t-test (ns – not significant).

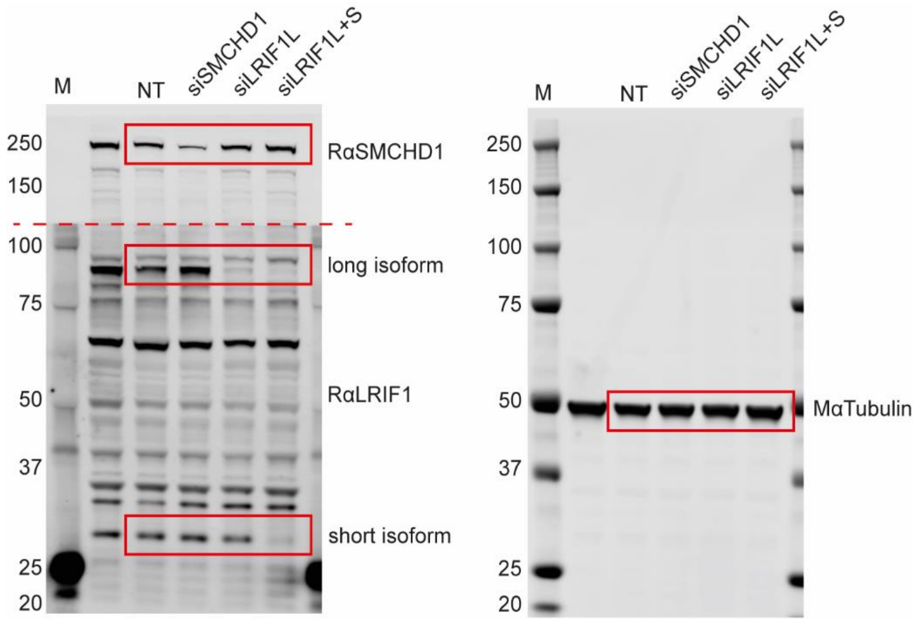

Supplementary Figure 7. Uncropped Western blot images corresponding to Figure 4b. Uncropped western blot for confirmation of successful siRNA-mediated knock-down of SMCHD1, LRIF1L or LRIF1L+S in primary ICF1 myoblasts. Tubulin was used as a loading control.

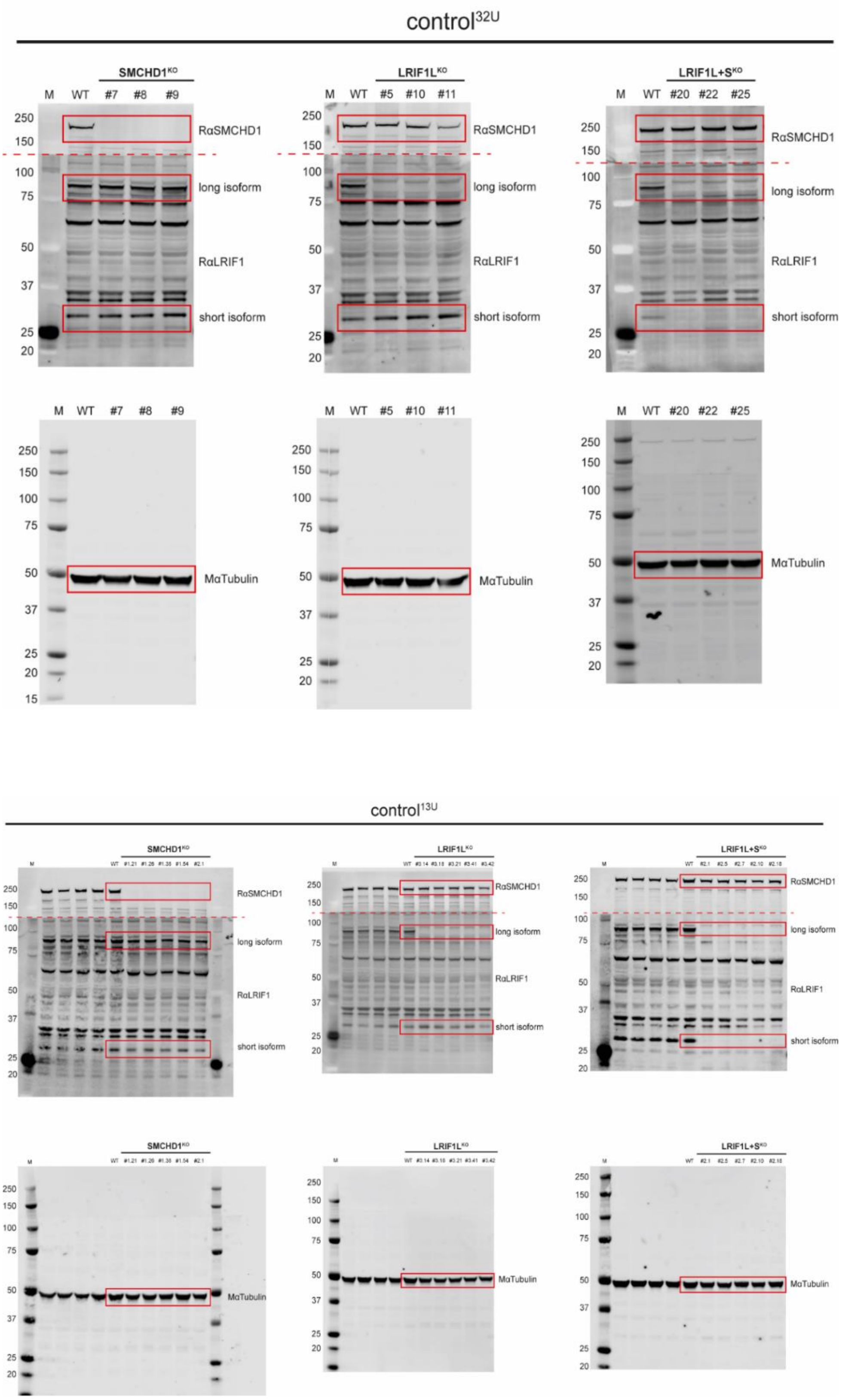

Supplementary Figure 8. Uncropped Western blot images corresponding to Figure 1a.Uncropped western blots for confirmation of successful SMCHD1 and LRIF1 knockouts in control32U and control13U myogenic cell lines. Tubulin was used as a loading control.

Supplementary Table 1. Additional information about cell lines used in this study. The percentage value in the Remarks columns refers to 4q+10q D4Z4 methylation measured in blood and expressed as the Delta1 score rounded to the nearest integer. The Delta1 score is determined by measuring methylation-sensitive FseI digestion efficiency with Southern blotting and adjusting the observed methylation for the cumulative size of both 4q and 10q repeats. For orientation, the average Delta1 in control individuals is almost 0, whereas for *SMCHD1* mutation carriers it is −31.3% as previously reported by Lemmers et al [26].

Cell Line ID	Cell Type	Clinical Status	Male (M) or Female (F)	Primary (P) or Immortalized (I)	Remarks
control[32U]	myoblast	healthy control	F	I	-4%
control[13U]	myoblast	healthy control	M	I	N.D.
2445	myoblast	FSHD2 (*SMCHD1* c.4347-236A>G)	M	I	-29%
Rf285.3	myoblast	ICF1 (*DNMT3B*, c.2421-11G>A, c.2421-11G>A)	M	I	-42%, 11U 4qA161S
2524	fibroblast	healthy control	F	P	19%
2397	fibroblast	healthy control	M	P	12%
2333	fibroblast	healthy control	M	P	4%
2440	fibroblast	FSHD2 (*SMCHD1* c.1302_1306delTGATA)	F	P	-25%
2332	fibroblast	FSHD2 (*SMCHD1* c.3274_3276+1del)	M	P	-36%
2337	fibroblast	FSHD2 (*SMCHD1* 1.2 Mb deletion)	F	P	-29%
Rf732 (v294)	fibroblast	FSHD2 (*DNMT3B* c.1579T>C)	M	P	-21%
Rf210 (b974)	fibroblast	Healthy control (*DNMT3B* c.2072C>T)	M	P	-29%
Rf699.3	fibroblast	ICF1 (*DNMT3B* c.1918G>C, c.1918G>C)	F	P	-46%
Rf286.3	fibroblast	ICF1 (*DNMT3B* c.2177T>G, c.1918G>C)	M	P	-37%
2374	fibroblast	Healthy control	F	P	-1%
2417	fibroblast	Healthy control	F	P	-7%
1926	myoblast	Healthy control	F	P	-4%
2333	myoblast	Healthy control	M	P	4% myoblast
2417	myoblast	Healthy control	F	P	-7%
2337	myoblast	FSHD2 (*SMCHD1* 1.2 Mb deletion)	F	P	-29%
2338	myoblast	FSHD2 (*SMCHD1* 1.2 Mb deletion)	M	P	-26%
2413	myoblast	FSHD2 (*SMCHD1* 1.2 Mb deletion)	M	P	-28%
2440	myoblast	FSHD2 (*SMCHD1* c.1302_1306delTGATA)	M	P	-25%
GM08714	fibroblast	ICF1 (*DNMT3B* c.1807G>A, c.2232-11G>A)	F	P	-34%
Rf614	fibroblast	ICF1 (*DNMT3B* c.2292G>T, c.2342_2343del)	F	P	-39%

HCT116	colon carcinoma	NA	M	NA	57%, First described here [70]
HCT116 DKO	colon carcinoma	Double knockout of *DNMT3B* and *DNMT1*	M	NA	-37%, First described here [70]

Supplementary Table 2. Oligonucleotides used for sgRNAs cloning into pX458 vector. The extra G (underlined) was added upstream of the sgRNA sequence if the sgRNA sequence itself did not start with one to ensure transcription from the U6 promoter. The sequence specific to the targeted DNA region is in bold.

**Name**	**5'->3'**
SMCHD1 ex3 sgRNA3 F	CACCG**ACTGATTGACCGACTGTAGC**
SMCHD1 ex3 sgRNA3 R	AAAC**GCTACAGTCGGTCAATCAGT**C
LRIF1 ex2 gRNA948 F	CACCG**TCGCGTCCCACTAGGATCGA**
LRIF1 ex2 gRNA948 R	AAAC**TCGATCCTAGTGGGACGCGA**C
LRIF1 ex3 gRNA153 F	CACCG**AATGGTCAGGAATTCGAGTA**
LRIF1 ex3 gRNA153 R	AAAC**TACTCGAATTCCTGACCATT**C

Supplementary Table 3. Primers used for RT-qPCR analyses. All primer pairs were used at Tm = 60°C. *GUSB* was used as a house-keeping gene.

**Name**	**5'->3'**
GUSB F	CTCATTTGGAATTTTGCCGATT
GUSB R	CCGAGTGAAGATCCCCTTTTTA
Dux4RT F2	CCCAGGTACCAGCAGACC
pLAM R4	TCCAGGAGATGTAACTCTAATCCA
hMYH3 F	TGATCGTGAAAACCAGTCCATTCT
hMYH3 R	TTGGCCAGGTCCCCAGTAGCT
TRIM43 F	ACCCATCACTGGACTGGTGT
TRIM43 R	CACATCCTCAAAGAGCCTGA
ZSCAN4 F	TGGAAATCAAGTGGCAAAAA
ZSCAN4 R	CTGCATGTGGACGTGGAC
MBD3L2 F	GCGTTCACCTCTTTTCCAAG
MBD3L2 R	GCCATGTGGATTTCTCGTTT
KHDC1L F	TGAATCAGGTGGGAGCACAG
KHDC1L R	CAATGCAGCGAAGGTACGTG
SMCHD1 ex47 F	CGACAGATTGTCCAGTTCCTC
SMCHD1 ex48 R	CCAATGGCCTCTTCTCTCTG
LRIF1 ex2/3 F	GTGTCCTCCAGAGCATAGAG
LRIF1 ex2/3 R	GCCATCTCATTATGGATCTTTGG
LRIF1 ex1/3 F	TCGCGTTGATCCATAATGAG
LRIF1 ex1/3 R	CACTCTTCAGATGTAATGCCT
LRIF1 ex3/4 F	GTTTATGGTGAAGGAAGGAGAG
LRIF1 ex3/4 R	ACCGGTGACATTAGCTTCC

Supplementary Table 4. Primers used for ChIP-qPCR analyses.

**Name**	**5'->3'**	**Note (Tm, reference)**
DR ChIP F2	GGCAGGGAGGAAAAGCGGTCC	60°C, this paper
DR ChIP R2	CTGTGAACCGCGCGGGTGAAG	
Q ChIP F	CCGCGTCCGTCCGTGAAA	65°C, [52]
Q ChIP R	TCCGTCGCCGTCCTCGTC	
Hox ChIP F	CGAGGACGGCGACGGAGAC	58°C, [52]
Hox ChIP R	ACCCTGTCCCGGGTGCCTG	
LRIF1 prom 679 F	AAGGTGACTGGCTCGCTAAA	60°C, this paper
LRIF1 prom 830 R	TTTATGATTGACCCCGGAAA	
GAPDH prom F	CTGAGCAGTCCGGTGTCACTAC	60°C, this paper
GAPDH prom R	GAGGACTTTGGGAACGACTGA	
