# Supplementary material for: SMCHD1 and LRIF1 converge at the FSHD-associated D4Z4 repeat and LRIF1 promoter yet display different modes of action
Source: Commun Biol. 2023 Jun 28;6:677. doi: 10.1038/s42003-023-05053-0 (PMC10307901; doi:10.1038/s42003-023-05053-0)
Supplement: Supplementary file 3 — Description of Additional Supplementary Files [file 42003_2023_5053_MOESM3_ESM.pdf]

Description of Additional Supplementary Files

File name: Supplemental Data 1
Description: Final list of differentially expressed genes in SMCHD1KO, LRIF1LKO or LRIF1L+SKO clones compared to WT clones derived from the control32U muscle cell line.

File name: Supplemental Data 2
Description: The source data behind the graphs in the paper.
